# Relationship between Early Maladaptive Schemas and DSM-5 Pathological Personality Traits from a Dimensional Diagnostic Approach

**DOI:** 10.1192/j.eurpsy.2024.213

**Published:** 2024-08-27

**Authors:** J. Major, A. Matúz, B. Gács, B. Birkás

**Affiliations:** Behavioural Sciences, University of Pécs, Medical School, Pécs, Hungary

## Abstract

**Introduction:**

In DSM-5 Section III, the Alternative Model for Personality disorders (AMPD), a dimensional approach for conceptualization and diagnosing complex character problems was introduced. Based on recent findings, AMPD aligns well with the theory of Young’s Schema Therapy (ST). ST seems to offer a valuable clinical framework that complements the empirically based AMPD, which is not built upon a certain theory of psychopathology.

**Objectives:**

The aim of the current study was to explore the association between early maladaptive schemas (EMSs), DSM-5 pathological personality traits and certain psychological symptoms to gain a better understanding of their relationship and highlight the connection points between AMPD and the theory of ST.

**Methods:**

A total of 490 Hungarian participants, including 98 males, took part in the cross-sectional research, with an average age of 26.9 (SD = 9.34). All participants completed the short form of Young’s schema questionnaire (YSQ-S3), the brief form of PID-5 (PID-5 BF) and the revised version of the Derogatis Symptom Checklist (SCL-90 R).

**Results:**

Results of a series of hierarchical regression analyses found that all five schema domains were able to predict psychological symptoms and DSM-5 pathological personality traits at a statistically significant level. Moreover, in accordance with our data, specific EMS patterns are associated with different psychological symptoms and pathological personality traits. Ultimately, we identified two EMSs, namely Negativity/Pessimism and Insufficient Self-control, which predicted all of our dependent variables.

**Conclusions:**

Our findings suggest that the relationship between EMSs and DSM-5 pathological personality traits goes beyond the established fact that EMSs, like any other indicators of personality problems are associated with psychopathological symptoms and traits. This is supported by the fact that we could link specific EMS patterns to the pathological personality traits and psychological symptoms that we investigated. We believe that our results contribute to the clinical utility of AMPD, by assisting the creation of schema profiles tailored to personality pathologies, thereby facilitate the diagnostic process and the development of schema - focused interventions. Furthermore, it seems that the identified EMSs, Negativity/Pessimism and Insufficient Self-control play a special role in relation to pathological personality traits and psychological symptoms and should be considered with particular emphasis in terms of risk group classification and vulnerability.

**Disclosure of Interest:**

None Declared

